# A study on the expression of apoptotic molecules related to death receptor and endoplasmic reticulum pathways in the jejunum of AFB_1_-intoxicated chickens

**DOI:** 10.18632/oncotarget.20333

**Published:** 2017-08-18

**Authors:** Zhixiang Zheng, Zhicai Zuo, Panpan Zhu, Fengyuan Wang, Heng Yin, Xi Peng, Jing Fang, Hengmin Cui, Caixia Gao, Hetao Song, Ping Ouyang, Yi Zhou, Song Zhao

**Affiliations:** ^1^ Key Laboratory of Animal Diseases and Environmental Hazards of Sichuan Province, College of Veterinary Medicine, Sichuan Agricultural University, Chengdu, Sichuan 611130, PR China; ^2^ College of Veterinary Medicine, Sichuan Agricultural University, Chengdu, Sichuan 611130, PR China; ^3^ College of Life Sciences, China West Normal University, Nanchong, Sichuan 637002, PR China; ^4^ Life science department, Sichuan Agricultural University, Yaan, Sichuan 625014, PR China

**Keywords:** aflatoxin B_1_, apoptosis, jejunum, death receptor molecules, endoplasmic reticulum molecules

## Abstract

Aflatoxin B_1_ (AFB_1_) is a common contaminant of poultry feeds in tropical and subtropical climates. Early researches have well established the hepatotoxic, carcinogenic, and immunotoxic effects of AFB_1_ on humans and animals. Recently, it has been shown that AFB_1_ could cause the up- or down-alteration of mitochondrial pathway molecule expression. However, the information on the expression of death receptor and endoplasmic reticulum molecules in the jejunal apoptosis induced by AFB_1_ were unavailable. So the present study was conducted to explore the expression of apoptotic molecules related to death receptor and endoplasmic reticulum in the jejunal cells of chickens exposed to AFB_1_ diet for 3 weeks. Total of 144 one-day-old chickens was randomly divided into two groups, namely control group (containing 0 mg/kg AFB_1_) and AFB_1_ group (containing 0.6 mg/kg AFB_1_). Histopathological observation and microscopic quantitative analysis revealed morphological changes in the jejunum such as the shedding of the mucosal epithelial cells in the apical region of villi along with the decrease of villus height, villus area and villus/crypt ratio in the AFB_1_ group. Both TUNEL and flow cytometry assays showed that AFB_1_ intake induced excessive apoptosis of jejunal cells. Quantitative real-time PCR test displayed the general upregulation of death receptors (FAS, FASL, TNF-α and TNF-R1), endoplasmic reticulum signals (GRP78 and GRP94) as well as initiator and executioner caspases (CASPASE-10, CASPASE-8 and CASPASE-3) in the jejunum of AFB_1_-intoxicated chickens. It's the first study demonstrating that AFB_1_ induced apoptosis of chickens’ jejunum accompanied by the alteration of death receptor and endoplasmic reticulum molecule expression.

## INTRODUCTION

Aflatoxins are the secondary metabolites of *Aspergillus flavus* and *Aspergillus parasiticus*. In humid areas, aflatoxins have the highest incidence in food and feed. So far, more than 20 kinds of aflatoxins have been isolated, including aflatoxin B_1_, B_2_, G_1_, G_2_ and so on [1]. Of these toxins, aflatoxin B_1_ (AFB_1_) is the most commonly encountered and is supposed to have higher toxicity than other aflatoxins [2]. International Agency for Research on Cancer (IARC) has produced sufficient evidences of carcinogenicity of AFB_1_ and classified it as a Group I human carcinogen [3]. Up to now, the hepatotoxic, carcinogenic, genotoxic, immunotoxic and other detrimental effects of AFB_1_ in many animal species including humans have been well documented [4–6].

Apoptosis is the process of programmed degradation and death, which aims to eliminate abnormal, senescent and harmful cells in the body, however, may also occurs as a response to various environmental stimuli including toxicity. Available information have shown that AFB_1_ worked as a direct or indirect initiator as well as promoter of apoptotic process [7, 8]. For instances, AFB_1_ caused apoptosis of hepatocytes [7, 9], thymocytes [10], splenocytes [11], bronchial epithelial cells [12], jejunal mucosal cells [13] and the bursa of Fabricius cells [14].

The gastrointestinal tract is responsible for digestion and absorption of food components [6]. As part of the small intestine, the jejunum accounts for a long part in small intestine and has a strong ability for absorption. Epithelium cells in the small intestine have a high turnover, and apoptosis is quite important for controlling the majority of intestinal epithelial cell loss [15–17]. Our early study showed that 0.3 mg/kg AFB_1_ in the chickens’ diet caused alteration of BAX, BCL-2, and CASPASE-3 expressions involved in apoptosis related to mitochondrial pathway in chickens’ jejunum [13]. It is well known that there are three key regulatory molecules involved in apoptosis: mitochondria, death receptor and endoplasmic reticulum (ER) signals [18]. However, the expression of death receptor and ER molecules in the jejunum of AFB_1_-intoxicated chickens have not been reported.

Therefore, the objectives of this study were to explore the expression of apoptotic molecules related to death receptor and endoplasmic reticulum in the jejunal cells by using a broiler model, based on the histopathological observation, microscopic quantitative analysis, TUNEL assay, along with flow cytometry and quantitative real-time PCR test. The results from the present study would provide a reference for the further study of the apopototic mechanism induced by AFB_1_ in jejunum, and may be helpful in bringing down the toxigenic potential of AFB_1_.

## RESULTS

### Pathological observation and microscopic quantitative analysis

**Figure 1:**
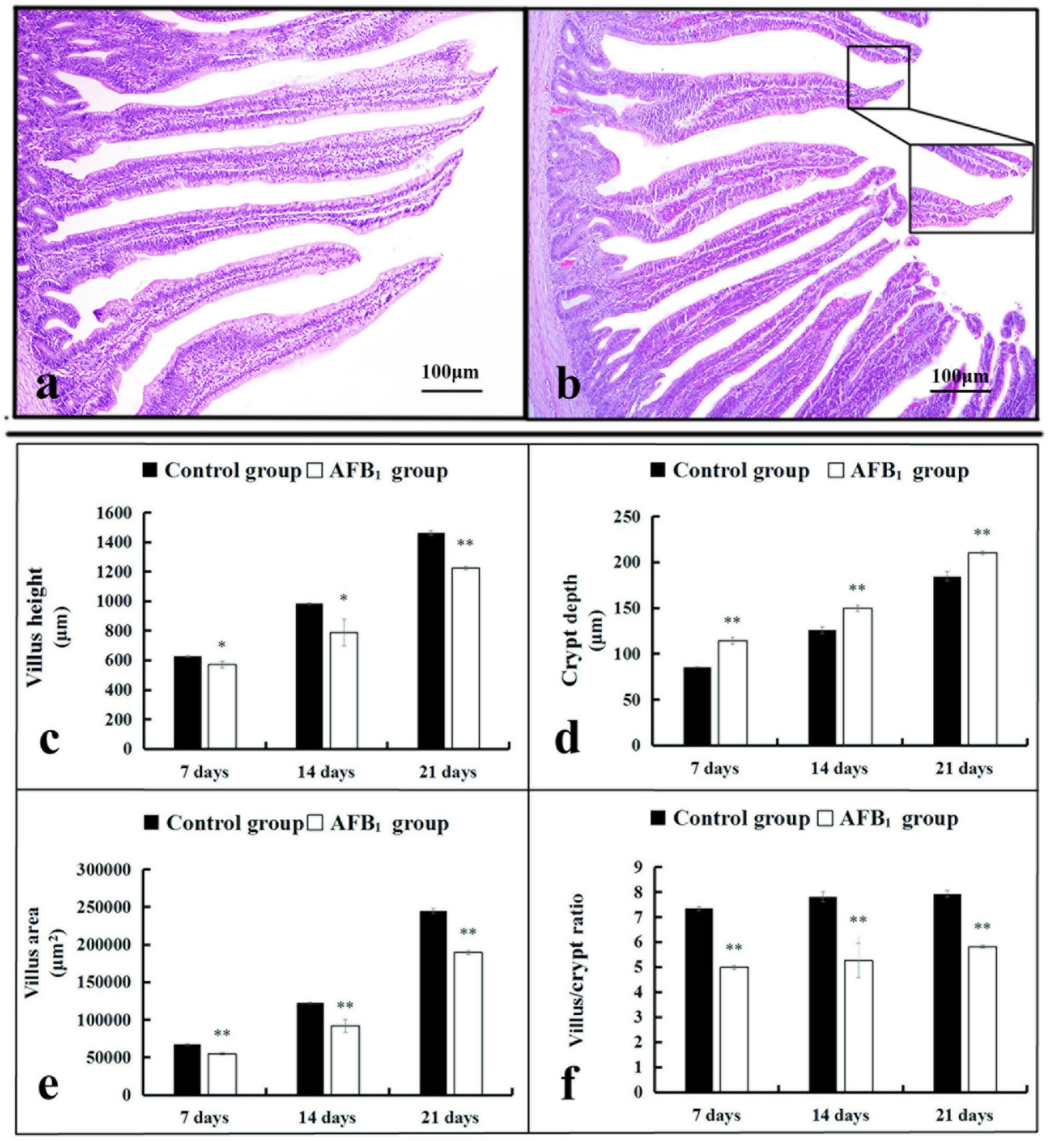
The histological structure of jejunum and the values of microscopic quantitative analysis **(a-b)** The histological structure of jejunum at 14 days of age (HE staining, scale bar: 100 μm), (a) control group; (b) AFB_1_ group. **(c-f)** The values of microscopic quantitative analysis, c-f: the values of villus height, crypt depth, villus area and villus/crypt ratio, respectively. Note: data are presented with the means ± standard deviation (n=6). ^*^p < 0.05, ^**^p < 0.01 compared with the control group.

Pathological observation showed that the mucosal epithelial cells in the apical region of villi were obviously shedding in the AFB_1_ group at 7, 14 and 21 days of age when compared with the control group (Figure 1a-1b). Microscopic quantitative analysis revealed that the villus heights, villus areas and villus/crypt ratios of the AFB_1_ group were significantly decreased (p<0.05 or p<0.01), whereas the crypt depths of the AFB_1_ group were significantly increased when compared with those of the control group during the experiment (p<0.01) (Figure 1c-1f).

### The jejunal cell apoptosis by flow cytometry assay

**Figure 2:**
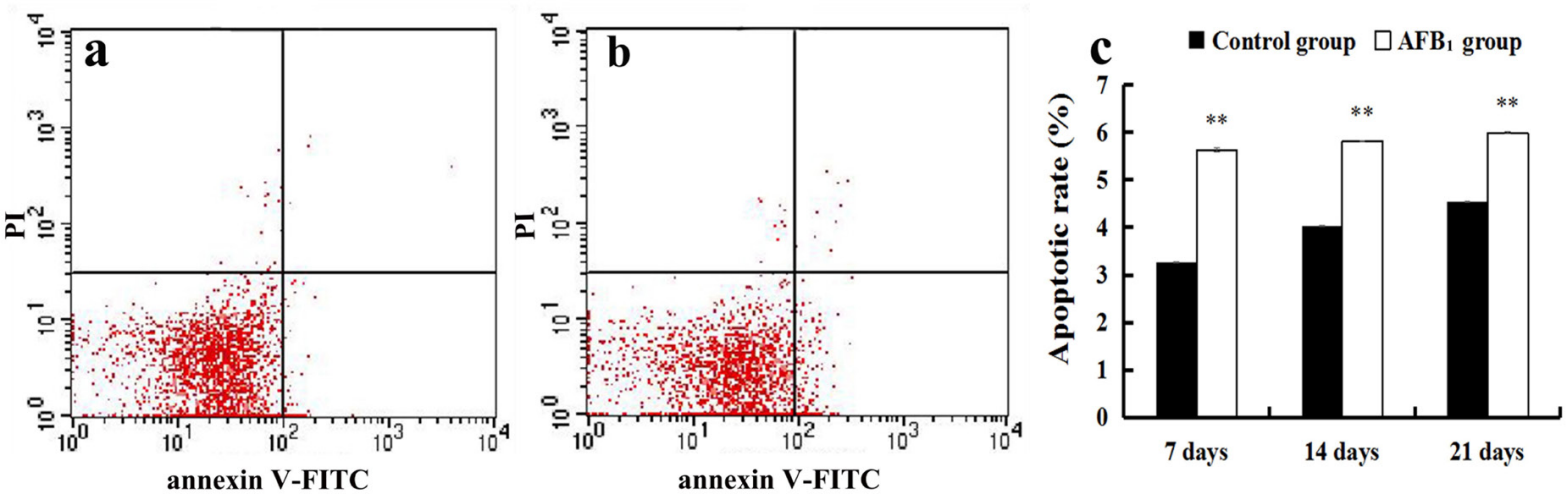
The jejunal cell apoptosis by flow cytometry assay **(a-b)** Scattergram of apoptotic jejunal cells obtained by flow cytometry assay at 21 days of age, (a) control group; (b) AFB_1_ group. **(c)** Apoptotic rates by flow cytometry assay. Note: data are presented with the means ± standard deviation (n=6). ^**^p < 0.01 compared with the control group.

The percentage of apoptotic cells was quantitatively detected by flow cytometry. Apoptotic cell counts were measured by detecting the total percentage of early (Annexin-V positive and PI negative) and late (both Annexin-V and PI positive) apoptotic cells. When compared with the control group, the percentages of apoptotic cells of the jejunal cells in the AFB_1_ group were significantly increased at 7, 14 and 21 days of age (p<0.01) (Figure 2a-2c).

### The jejunal cell apoptosis by TUNEL assay

**Figure 3:**
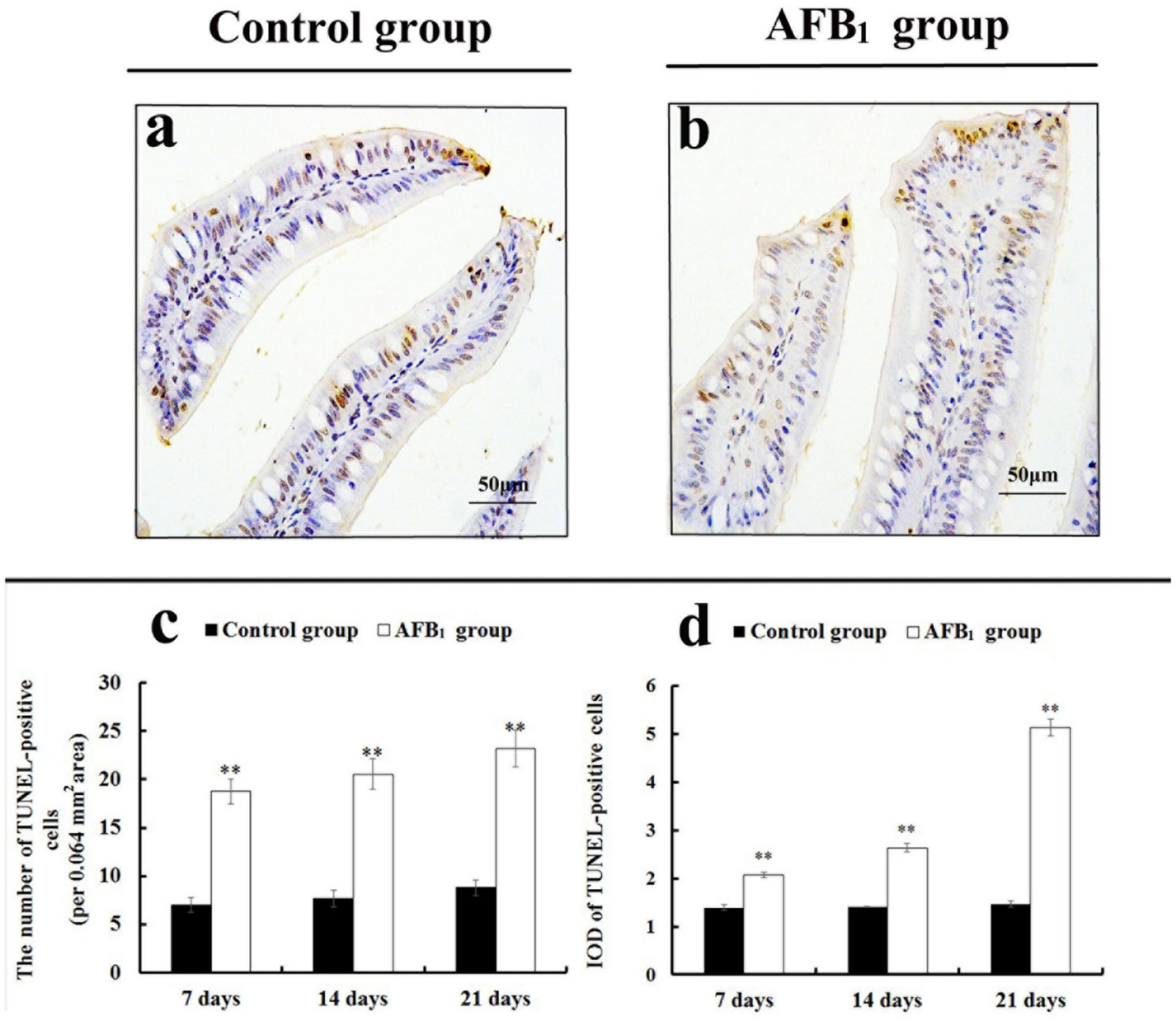
The jejunal cell apoptosis by TUNEL assay **(a-b)** TUNEL-positive cells in the apical regions of jejunal villi at 21 days of age (TUNEL assay, scale bar: 50 μm), (a) control group; (b) AFB_1_ group. **(c)** The number of TUNEL-positive cells. **(d)** The integrated optical density (IOD) of TUNEL-positive cells. Note: data are presented with the means ± standard deviation (n=6). ^**^p < 0.01 compared with the control group.

TUNEL assay demonstrated that the nuclei of TUNEL-positive cells were stained brown in two groups (Figure 3a-3b). These positive cells were mainly distributed in the apical region of villus. Compared with the control group, more TUNEL-positive cells were observed in the AFB_1_ group (Figure 3a-3b). Furthermore, microscopic quantitative analysis revealed that both the number and integrated optical density of TUNEL-positive cells in the AFB_1_ group were significantly increased at 7, 14, and 21 days of age, in comparison to the control group (Figure 3c-3d).

### Expression levels of apoptotic regulatory mRNAs in the jejunal cells by qRT-PCR

**Figure 4:**
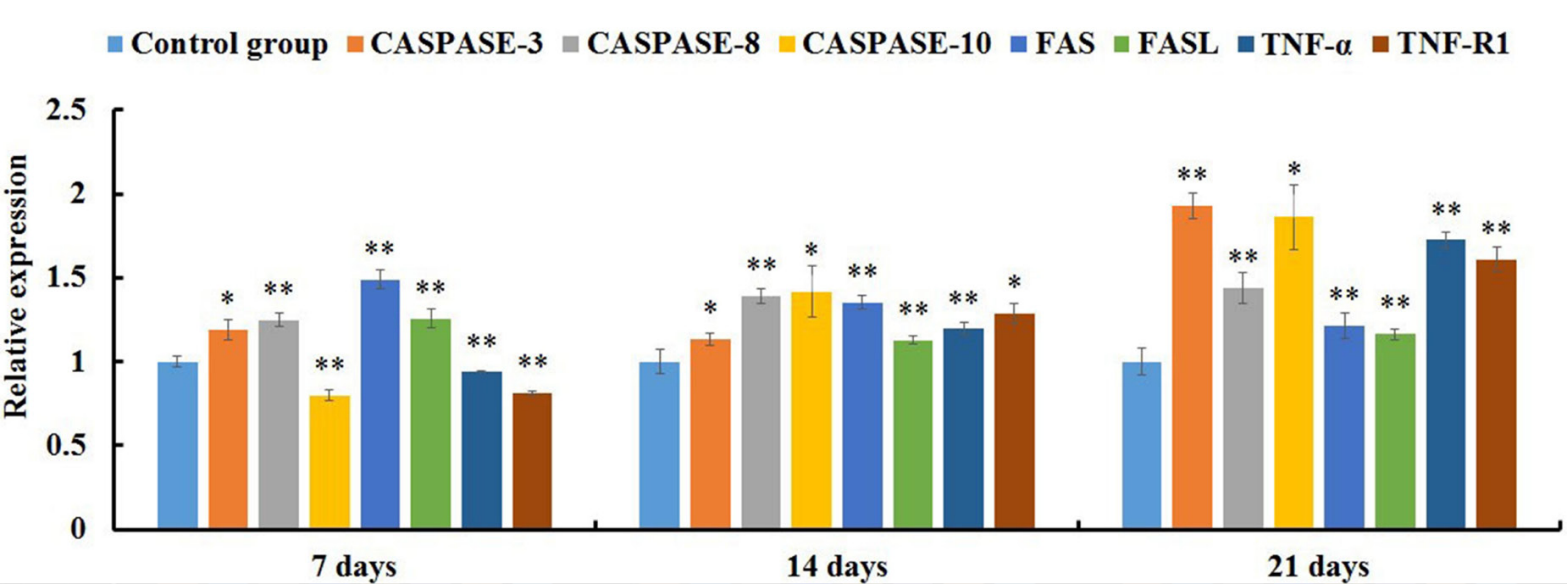
The expression levels of mRNAs involved in the death receptor pathway of the jejunal cell apoptosis of the AFB1-fed chicken and expressed as fold change relative to the control group Note: data are presented with the means ± standard deviation (n=6). ^*^p < 0.05, ^**^p < 0.01 compared with the control group.

**Figure 5:**
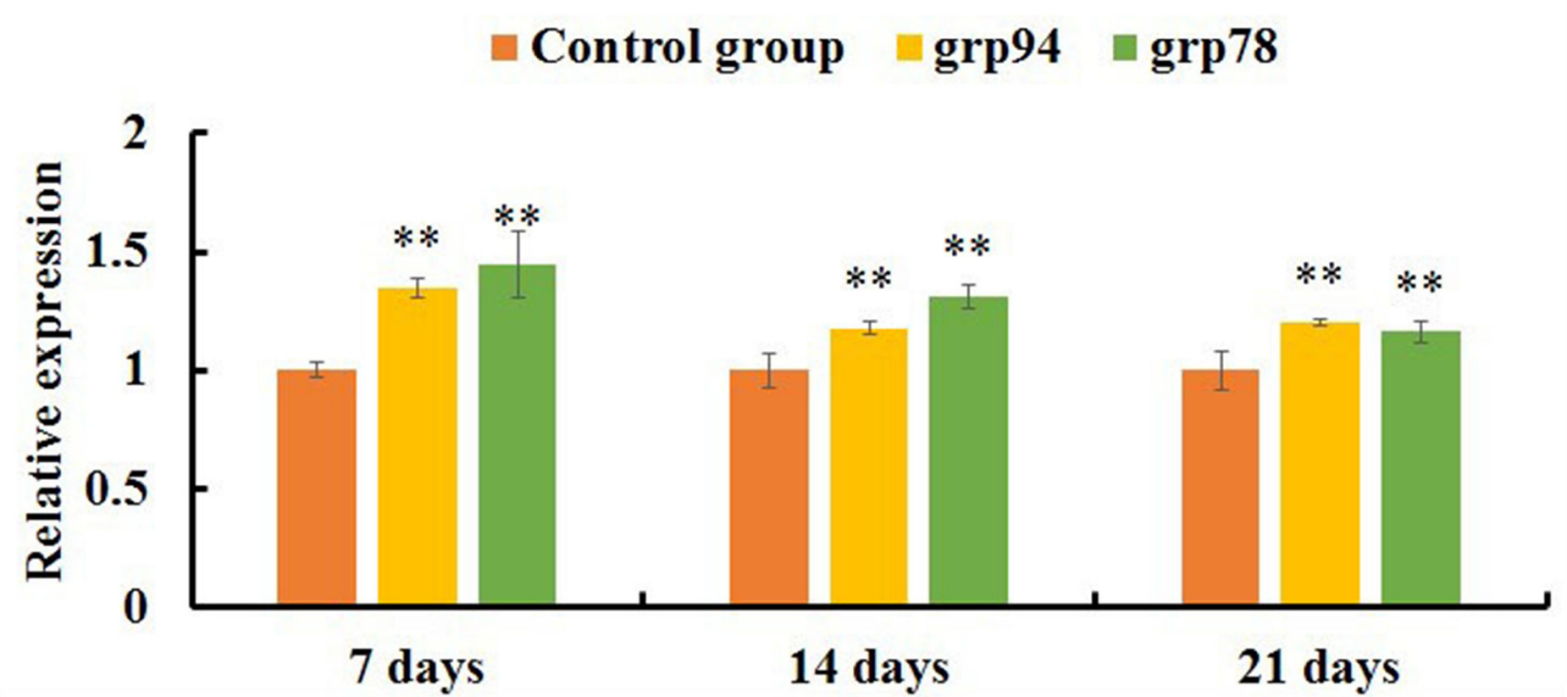
The expression levels of mRNAs involved in the ER pathway of the jejunal cell apoptosis of the AFB1-fed chicken and expressed as fold change relative to the control group Note: data are presented with the means ± standard deviation (n=6). ^*^p < 0.05, ^**^p < 0.01 compared with the control group.

qRT-PCR analysis showed that the expression levels of FAS, FASL, CASPASE-8 and CASPASE-3 mRNAs were significantly increased in the AFB_1_ group at 7, 14 and 21 days of age (p<0.05 or p<0.01) when compared with the control group (Figure 4). The expression levels of TNF-α, TNF-R1 and CASPASE-10 mRNAs in the AFB_1_ group were significantly higher than those in the control group (p<0.01) except for 7 days of age when these values were significantly lower than those in the control group (p<0.01) (Figure 4). Furthermore, compared with the control group, the expression levels of GRP78 and GRP94 mRNAs in the AFB_1_ group were significantly increased at 7, 14 and 21 days of age (p<0.01) (Figure 5).

## DISCUSSION

The small intestinal epithelium is a rapidly renewing tissue, in which cells are lost from the villus into the gut lumen and are generally replaced at an equal rate by the proliferation of cells in the crypts [16]. The intestinal villus is to enlarge the absorptive areas and to promote digestion and absorption. Thus, the measurement of the villus height, crypt depth, and villus height to crypt depth ratio is a well-known way to investigate the intestinal function [19]. However, measuring the villus height by itself does not take into account the variations which may occur because of variational villus diameter. To avoid this, in the present study the villus area was also measured. Furthermore, it has been shown that apoptosis also plays an important role in the villus growth [15–17, 20, 21]. TUNEL assay can identify DNA fragmentation and examine the topographic distribution of apoptotic cells. Measuring the number and integrated optical density of TUNEL-positive reaction are the microscopic quantitative analyses to evaluate apoptotic level under microscope. Moreover, flow cytometry assay is an effective way to detect early and late stages of apoptotic cells. Our present results showed that AFB_1_ induced histopathological injury of jejunum including the shedding of the absorptive cells in the villus tip and the reduction of the villus height, villus area and villus height/crypt ratio, and caused excessive apoptosis based on the TUNEL and flow cytometry assays, in line with our early report [22]. These results also indicated that the increased apoptosis and the shedding of the absorptive cells should be responsible for the retarded villus growth of jejunum invoked by AFB_1_.

Apoptosis is a highly regulated cell death program and is triggered through the mitochondria, death receptor and ER pathways. Early study has shown that the mitochondria pathway including BCL-2 and BAX genes were related to the excessive apoptosis of jejunum provoked by AFB_1_ [13]. To fully understand the alteration of AFB_1_-induced apoptotic associated genes in the jejunum, the expression of death receptor and ER molecules was further explored.

The death receptor pathway can be induced by the activation of death receptors including FAS, FASL, TNF-α, TNF-R1 and CASPASES. FAS is a 45-kD type I membrane receptor that is a member of the tumor necrosis factor family of surface receptors [23, 24]. FASL is a 37-kD type II membrane glycoprotein that belongs to a member of the tumor necrosis factor family of cytokines [25]. Trimerization of the FAS receptor by FAS ligand results in activation of CASPASE-8 and subsequently stimulates the activation of down-stream CASPASES, including CASPASE-3, leading to cell death [26]. TNF-α, a pleiotropic proinflammatory cytokine, has a role in inducing cell apoptosis. TNF-α exerts its biological activity by binding to type 1 and type 2 receptors (TNF-R1 and TNF-R2). TNFRs belong to a large family of nerve growth factor receptors/TNFRs [27–29]. TNF-R1 is a transmembrane receptor with one to five cysteine-rich repeats in their extracellular domains and a common deathdomain (DD) in their cytoplasmic tail [30]. Upon ligation with TNF-α, TNF-R1 undergoes trimerization of its receptor. TNF-R-associated death domain (TRADD), which in turn recruits another adapter molecule, the Fas-associated death domain (FADD). FADD recruits PROCASPASE-8 by protein-protein interaction via homologous death effector domain (DED) to form a death inducing signal complex (DISC) [31, 32]. During DISC formation, PROCASPASE-8 is autolytically cleaved to yield active CASPASE-8. Active CASPASE-8 is rapidly released from the DISC to cytoplasm and serves as an enzyme for downstream effector CASPASE-3, CASPASE-6, and CASPASE-7. CASPASE-10 shares homologous DEDs with CASPASE-8, suggesting that CASPASE-10 may also function by interacting with death receptors [33]. These effector CASPASES (especially CASPASE-3) cleaves a number of substrates resulting in morphologic and biochemical features of apoptosis [34, 35]. The present study demonstrated that AFB_1_ generally induced over expression of FAS, FASL, TNF-α, TNF-R1 along with CASPASE-3, CASPASE-8 and CASPASE-10 mRNAs in the jejunum, suggesting that the death receptor molecules may involve in the excessive apoptosis of jejunal cells. Similar results were also revealed in the apoptosis of hepatocytes and thymocytes of chicken provoked by AFB_1_ [36, 37]. Contrary to the above results, the expression levels of FAS, FASL, FADD, CASPASE-8 and CASPASE-10 mRNAs of the bursa of Fabricius cells in the AFB_1_ group were not significantly different from those in the control group, indicating that the death receptor pathway may not contribute to the excessive cell death of the bursa of Fabricius cells induced by AFB_1_ [14]. Thus, the signaling pathways involved in the AFB_1_-caused apoptosis were not conclusive, which might be attributed to the different tissues or cells.

In the ER pathway, apoptosis mainly occurs through the ER stress. GRP78 and GRP94 (Glucose Regulated Protein 78 and 94 kDa) are heat shock protein family molecular chaperones that are found in the lumen of the ER. They are essential regulators of ER function due to their function in protein translocation, folding and assembly, targeting misfolded proteins for degradation, ER Ca^2+^ binding and controlling the initiation of ER stress sensors [38]. ER stress is a phenomenon that develops either due to accumulation of unfolded or misfolded proteins within the ER or a Ca^2+^ store depletion [39]. Accumulation of unfolded proteins in the lumen of the ER induce unfolded protein response (UPR). During this process, the molecular chaperones (such as GRP78, GRP94) are activated and their expressions maintain proteins folding and eliminate the misfolded proteins. If the cells do not deal with accumulated misfolded proteins, a long term ER stress triggers apoptosis by recruiting the proapoptotic members of the BCL-2 family to the ER surface, and activating CASPASE-12, finally activating CASPASE-3 [39, 40]. Therefore, GRP78 and GRP94 play an important role in the cell apoptosis triggered by the ER pathway. In the present study, the expression levels of GRP78 and GRP94 mRNAs were both significantly up-regulated in the AFB_1_ group when compared with the control group. Furthermore, our early study has demonstrated that AFB_1_ could induce the up-regulation of BAX mRNA expression and down-regulation of BCL-2 mRNA expression associated with the decrease of BCL-2/BAX ratio in the broilers’ jejunum [13]. Thus, it is tempting to speculate that the ER molecules may be involved in the AFB_1_-induced excessive jejunal apoptosis. This is in line with the report in the bursa of Fabricius cells [14]. However, contradictory results showed that the ER pathway may not involve in the AFB_1_-induced apoptosis of thymocytes [37]. This is also confirmed that the apoptotic signaling pathways provoked by AFB_1_ may vary depending on different tissues.

## CONCLUSION

It is concluded that 0.6 mg/kg AFB_1_ in the diet could cause histopathological changes and induce apoptosis in the jejunal cells of broilers, which was accompanied by the general increase of FAS, FASL, TNF-α, TNF-R1, CASPASE-3, CASPASE-8, CASPASE-10, GRP78 and GRP94 mRNAs expression.

## MATERIALS AND METHODS

### Experimental diet

Total of 144 one-day-old healthy Cobb chicken broilers was purchased from Chia Tai Group (Wenjiang, Sichuan, China), and randomly divided into control group (0 mg/kg AFB_1_ of basal diet) and AFB_1_ group (0.6 mg/kg AFB_1_ of basal diet) with three replicates per group and 24 birds per replicate. AFB_1_ was purchased from Sigma-Aldrich (USA, A6636). The basal diet, namely the control diet, was formulated according to National Research Council (NRC, 1994) [41] and Chinese Feeding Standard of Chicken (NY/ T33-2004) recommendations. The AFB_1_-contaminated diet was made, according to the method described by Kaoud [42]. Briefly, 27 mg AFB_1_ was dissolved into 30 ml methanol, then the 30 ml mixture was added into 45 kg corn-soybean basal diet to make up the AFB_1_-contaminated diet which contained 0.6 mg/kg AFB_1_. The equivalent methanol was added into corn-soybean basal diet to formulate control diet. Then the methanol of diets was evaporated at 98 °F (37 °C). The AFB_1_ concentration was analyzed by HPLC (Waters, Milford, MA, USA) with fluorescence detection (Waters, Model 2475, Milford, MA, USA), and the AFB_1_ concentration was determined as <0.001 mg/kg and 0.601 mg/kg in the control diet and AFB_1_ diet, respectively. Chickens were fed in cages with electrically heated units and provided with water as well as aforementioned diet *ad libitum* for 21 days. The animal protocols used in this work and all procedures of the experiment were performed based on the laws and guidelines of Sichuan Agricultural University Animal Care and Use Committee (Approval No: 2012-024).

### Histopathological observation and microscopic quantitative analysis

During the period of experiment, six broilers in each group were randomly chosen and euthanized at 7, 14 and 21 days of age. And jejunum (the midpoint between the bile duct entry and Meckel's diverticulum) was immediately fixed in 4% paraformaldehyde. After 24 h for fixation, tissues were dehydrated in alcohol, embedded by paraffin, sectioned at 5 μm, and stained with haematoxylin and eosin (HE) for histological observation. The histological structures of the tissues were observed and photographed with a digital camera (Nikon, DS-Ril, Japan). Microscopic quantitative analysis was carried out as follows: altogether ten measurements were taken per broiler for each parameter in the jejunum stained with HE using Image-Pro Plus 5.1 (USA) image analysis software. The following parameters were determined: villus height (the length from the top of the villus to the crypt mouth), villus area {the villus height multiplying villus width (the width of the middle of the villus)}, crypt depth (the length from the crypt mouth to the crypt base following the crypt lumen) and ratio of the villus height and crypt depth (villus/crypt).

### Cell apoptosis analysis by flow cytometry

At 7, 14, and 21 days of the experiment, six broilers in each group were euthanized, and jejuna were sampled from each chicken to determine the percentage of apoptotic cells by flow cytometer, similar to the method reported by Chen [43]. Briefly, the dissected jejuna were thereupon homogenized to form a cell suspension and filtered, then the cells were washed and resuspended in phosphate buffer at a concentration of 1×10^6^ cells/mL. 5 μL Annexin V-Fluorescein isothiocyanate (V-FITC) and 5 μL propidium iodide (PI) were added into 100 μL cell suspension, and incubated at 25 °C for 15 min in the dark. 400 μL 1× Annexin binding buffer was added to the mixture, and then the apoptotic cells were assayed by flow cytometer (BD FACSCalibur) within 1 h. The annexin V-FITC Kit was obtained from BD Pharmingen (USA, 556547).

### TUNEL assay

TUNEL assay was carried out according to the manufacturer's instruction of the Apoptosis Detection Kit (Boster Corporation). Briefly, the jejunal paraffin sections were dewaxed with 100% xylene, and rehydrated in successive changes of 100%, 95%, 85% and 75% ethanol. After endogenous peroxidase activity was quenched for 10 min in 3% H_2_O_2_ with distilled water at 37 °C, the sections were incubated with proteinase K diluted 1:200 in TBS at 37 °C for 5-10 min in a humid chamber. A labeling mixture containing digoxin-dUTP in Terminaldeoxynucleotidyl Transferase (TdT) enzyme buffer was added to the sections and incubated at 37 °C for 2 h. After three successive washings with TBS for 2 min, sections were covered with anti-digoxin-biotin conjugate diluted 1:100 in blocking regent and incubated for 30 min at 37 °C. The tissues were then incubated for 1 h at 37 °C with streptavidin-biotin-complex (SABC) diluted 1:100 in TBS. Labeling was visualized with 3’3’-diaminobenzidene. The sections were then counterstained with haematoxylin. For the negative control, representative sections were processed in the same way, while incubation with TdT enzyme buffer was omitted.

The number and integrated optical density (IOD) of TUNEL-positive cells were evaluated as following method. Briefly, photographs of TUNEL staining were taken with a digital microscope camera system (Nikon DS-Ri1, Japan). For each section, five fields of 0.064 mm^2^ from each area of the image (corresponding to five fields at 400× magnification) were analyzed using computer-assisted image-Pro Plus 5.1 (USA) image analysis software. By selecting ‘colour-chosen target’ in the option bar of the morphologic analysis system, all TUNEL-positive cells in the field were marked in colour. Then, ‘calculating’ in the option bar was selected to automatically calculate the number and IOD values.

### Quantitative real-time PCR (qRT-PCR) analysis

The jejunal mucosae from six broilers in each treatment at 7, 14, and 21 days of the experiment were stored in liquid nitrogen, respectively. Adding liquid nitrogen, the jejunal mucosae were crushed with pestle to homogenize until powdery, respectively. Total RNA was extracted from the powdery of jejunal mucosae using RNAiso Plus (9108/9109, Takara, Otsu, Japan). The mRNA was then reversely transcribed into cDNA using Prim Script™ RT reagent Kit with gDNA Eraser (RR047A, Takara, Otsu, Japan). The cDNA was used as a template for quantitative real-time PCR analysis.

For qRT-PCR reactions, 25 μL mixtures were made by using SYBR^®^ Premix Ex Taq™ II (DRR820A, Takara, Otsu, Japan), containing 12.5 μL Tli RNaseH Plus, 1.0 μL of forward and 1.0 μL of reverse primer, 8.5 μL RNAase-free water and 2 μL cDNA. Reaction conditions were set to 3 min at 95 °C (first segment, one cycle), 10 s at 95 °C and 30 s at Tm of a specific primer pair (second segment, 44 cycles) followed by 10 s at 95 °C, and 72 °C for 10 s (dissociation curve segment) using Thermal Cycler (C1000, BIO RAD, CA, USA). The expression of FAS, FASL, TNF-α, TNF-R1, GRP78, GRP94, CASPASE-3, CASPASE-8 and CASPASE-10 mRNAs was analyzed, and β-actin was used as an internal control gene. Sequence of primers was obtained from GenBank of NCBI. Primers were designed with Primer 5, and synthesized by BGI Tech (Shenzhen, China) (Table 1). The control broilers responses (mRNA amount) were been as reference values for between treatments comparisons within the same control day in each week, respectively. The results were analyzed with 2^-ΔΔCt^ calculation method [44].

**Table 1:** Primer sequences, corresponding accession numbers and sizes of the amplification products

Gene	Primer	Sequences(5’-3’)	Product size(bp)	Accession number
CASPASE-3	F	TGGCCCTCTTGAACTGAAAG	139	NM_204725
	R	TCCACTGTCTGCTTCAATACC		
CASPASE-8	F	GTCTCCGTTCAGGTATCTGCT	143	NM_204592
	R	TCTCAATGAAAACGTCCGGC		
CASPASE-10	F	CTGGGGGCTCCAAAAGTCC	204	XM_421936
	R	AAAGGGGGACAAAGCCAACA		
FAS	F	TCCACCTGCTCCTCGTCATT	78	NM_001199487
	R	GTGCAGTGTGTGTGGGAACT		
FASL	F	GGCATTCAGTACCGTGACCA	78	NM_001031559
	R	CCGGAAGAGCACATTGGAGT		
GRP78	F	GGTGTTGCTTGATGTGTGTCC	134	NM_205491
	R	GCTGATTGTCAGAAGCTGTGG		
GRP94	F	TGACCTGGATGCAAAGGTGGA	250	NM_204289
	R	TTAAACCCCACACCATCCCTCAAC		
TNF-α	F	TCAGACCAGATGGGAAGGGA	127	AY765397
	R	ACTGGGCGGTCATAGAACAG		
TNF-R1	F	CCTGTCTGTCTTCCCTGTCC	120	NM_001030779
	R	GGTGCATGGGGTCTTTTCTA		
β-actin	F	TGCTGTGTTCCCATCTATCG	178	L08165
	R	TTGGTGACAATACCGTGTTCA		

### Statistical analysis

The significance of difference between two groups was analyzed by variance analysis, and the results were expressed by means ± standard deviation. The analyses were performed using the independent sample test of SPSS 20.0 software (IBM Corp, Armonk, NY, USA) for windows. Statistical significant differences were considered at p <0.05 and markedly significant differences were considered at p< 0.01.
